# Detection of *Scedosporium* spp.: Colonizer or pathogen? A retrospective analysis of clinical significance and management in a large tertiary center

**DOI:** 10.1093/mmy/myae002

**Published:** 2024-01-18

**Authors:** Ilana Reinhold, Chantal Quiblier, Frank Blaser, Jan Bögeholz, Frank Imkamp, Macé M Schuurmans, Michael B Soyka, Reinhard Zbinden, Nicolas J Mueller

**Affiliations:** Department of Infectious Diseases and Hospital Epidemiology, University Hospital Zurich, Zurich, Switzerland; Institute of Medical Microbiology, University of Zurich, Zurich, Switzerland; Department of Ophthalmology, University Hospital Zurich, Zurich, Switzerland; Department of Medical Oncology and Hematology, University Hospital Zurich, Zurich, Switzerland; Institute of Medical Microbiology, University of Zurich, Zurich, Switzerland; Division of Pulmonology, University Hospital Zurich, Zurich, Switzerland; Department of Otorhinolaryngology, Head and Neck Surgery, University Hospital Zurich, Zurich, Switzerland; Institute of Medical Microbiology, University of Zurich, Zurich, Switzerland; Department of Infectious Diseases and Hospital Epidemiology, University Hospital Zurich, Zurich, Switzerland

## Abstract

Infections with *Scedosporium* spp. are emerging in the past two decades and are associated with a high mortality rate. Microbiological detection can be associated with either colonization or infection. Evolution from colonization into infection is difficult to predict and clinical management upon microbiological detection is complex. Microbiological samples from 2015 to 2021 were retrospectively analyzed in a single tertiary care center. Classification into colonization or infection was performed upon first microbiological detection. Clinical evolution was observed until July 2023. Further diagnostic procedures after initial detection were analyzed. Among 38 patients with microbiological detection of *Scedosporium* spp., 10 were diagnosed with an infection at the initial detection and two progressed from colonization to infection during the observation time. The main sites of infection were lung (5/12; 41.6%) followed by ocular sites (4/12; 33.3%). Imaging, bronchoscopy or biopsies upon detection were performed in a minority of patients. Overall mortality rate was similar in both groups initially classified as colonization or infection [30.7% and 33.3%, respectively (*P* = 1.0)]. In all patients where surgical debridement of site of infection was performed (5/12; 42%); no death was observed. Although death occurred more often in the group without eradication (3/4; 75%) compared with the group with successful eradication (1/8; 12.5%), statistical significance could not be reached (*P* = 0.053). As therapeutic management directly impacts patients’ outcome, a multidisciplinary approach upon microbiological detection of *Scedosporium* spp. should be encouraged. Data from larger cohorts are warranted in order to analyze contributing factors favoring the evolution from colonization into infection.

Abbreviations ABPM: allergic bronchopulmonal mycosis
AF: antifungal
AFST: antifungal susceptibility testing
BAL: bronchoalveolar lavage
CF: cystic fibrosis
COPD: chronic obstructive pulmonary disease
ENT: ear nose and throat
ITS: inter spacer region
MIC: minimal inhibitory concentration
PCA: potato carrot agar
TBS: tracheobronchial secretion
TPL: transplantation

## Introduction

Fungal infections caused by dematiaceous moulds of the genus *Scedosporium* are caused by different species belonging to the *Scedosporium apiospermum* complex (including amongst others *Scedosporium apiospermum* and *Scedosporium boydii)* and *Scedosporium aurantiacum*, which can be found in soil and polluted water.^1^ The separation from the genus *Lomentospora* (formerly included to the genus of *Scedosporium*) is clinically highly relevant due to different drug resistance patterns.^2,3^ Clinical classification ranges from colonization to localized infections to invasive disease. Invasive disease with dissemination typically occurs in immunocompromised patients.^4^ Localized infections are associated with inoculation trauma in immunocompetent patients and are often described in eye trauma.^5,6^*Scedosporium* spp. interpreted as colonization is mostly linked to respiratory specimens of patients with cystic fibrosis (as the second most frequent fungus after *Aspergillus* spp.) or other chronic respiratory disease with perturbation of airway architecture and mucosal surface, including the ear, nose, and throat (ENT) area.^1,7–10^ Due to the increasing numbers of immunocompromised patients, infections with *Scedosporium* spp. have been emerging over the last two decades.^7,11,12^

The progression from colonization to infection in immunocompetent and immunocompromised hosts is not fully predictable. Typical risk factors for invasion are hematological stem cell transplantation (HSCT), solid organ transplantation (SOT), and diabetes mellitus.^1,13,14^ Furthermore, the evolution of colonization is only partially predicted by antifungal prophylaxis: some lung transplant recipients colonized with *Scedosporium* spp. will develop invasive breakthrough illness even while being on universal antifungal prophylaxis active against *Scedosporium* spp., while other patients on immunosuppressive treatment do not develop any infection despite lacking antifungal prophylaxis.^15–17^ Data on the evolution from colonization to infection under immunosuppressive treatment are scarce as most patients are under universal antifungal prophylaxis.

The diagnostic approach depends on microbiological culture and, if possible, on histopathology to further distinguish infection from colonization.^17^ Ideally, imaging of the suspected primary site of infection with evaluation for additional sites of infection should be performed.^18–20^

Treatment options are limited, as *Scedosporium* spp. may exhibit resistance to multiple treatment options, especially to Amphotericin B if used as single therapy.^7,16^ Recommended first-line treatment is voriconazole in conjunction with surgical excision in localized infections. Additionally, reduction of immunosuppressive therapy is advised.^4,16,17^

In this retrospective observational study, we aimed to analyze the microbiological detection of *Scedosporium* spp. in a large tertiary center over 6 years, including the clinical evolution from colonization to infection and related outcome. Diagnostic and therapeutic management, as well as the factors influencing the outcome of patients with infection were collected from the medical chart.

## Methods

From 2015 to 2021, all microbiological samples positive for *Scedosporium* spp. at the Institute of Medical Microbiology in Zurich, Switzerland with a signed general consent form from the University Hospital Zurich were eligible for further evaluation. The study was approved by the local ethics commission of Zurich (BASEC Nr. 2022-01090).

Patient's data were analyzed in the electronic hospital health record system. At initial microbiological detection, a classification of *Scedosporium* spp. colonization or infection was performed. Colonization was defined as the presence of *Scedosporium* spp. without any clinical evidence of infection. Infection was defined according to the revised EORTC criteria of 2019 for invasive fungal infections, including histopathological evidence of hyphae in otherwise non-infected tissue or as per physician's assessment according to clinical presentation and/or imaging modalities, as the EORTC mentioned host factors for mould infections are not applicable owing to the fact that infection can also affect healthy hosts.^21^ Infection was further subdivided into ‘localized infection’, ‘invasive infection’ according to an extension to surrounding tissue or ‘disseminated infection’ in case of positive blood cultures or infection at ≥ 2 non-contiguous sites.^10,22^

Additional initial diagnostic investigations, such as biopsy or imaging modalities aiming to differentiate infection from colonization at the time of microbiological detection were re-assessed.

Period of observation was until July 2023 or until date of death, respectively. Clinical evolution over time was classified into ‘no evolution’ or ‘from colonization to infection’. In patients with recurrent microbiological detection, days of recurrence were calculated at least 14 days following initial detection. Detection after less than 14 days was not considered as recurrent. Repetition of sampling were performed individually by the treating physicians and did not follow a standardized protocol.

Treatment modalities were subdivided into antifungal therapy (combined or single), concomitant reduction of immunosuppressive treatment and/or surgical debridement.^18^

Outcome was investigated for findings on imaging, occurrence of death, or eradication. Findings on imaging were classified into progression of infection, stable response, and regressive findings at six and 12 weeks after initial diagnosis, respectively.^23^ Days between first microbiological detection and death were calculated and cause of death was analyzed whether *Scedosporium* spp. infection was directly attributable or not. Eradication was assessed by negativity of repeated microbiological sampling and regression of clinical and imaging findings.

For the identification of filamentous fungi, clinical samples were analyzed by microscopy and cultured on different agar types as described previously.^24^ Briefly, samples were cultured on Sabouraud agar with gentamicin and chloramphenicol (Becton Dickinson AG, Switzerland) at 25°C and 35°C, on Mycosel agar (Becton Dickinson AG, Switzerland), and potato carrot agar (PCA), both at 25°C. Phenotypic identification up to genus level was based on macro- and micro-morphological criteria, according to de Hoog G.S., 2009.^25^

Molecular identification was done if it was deemed clinically relevant, if macro- and micro-morphological identification was not possible or when a respiratory specimen originated from a cystic fibrosis (CF) patient. Chromosomal DNA were extracted from isolates using the InstaGene extraction matrix (Biorad) according to the manufacturer's instructions. Identification was performed by amplification and sequencing of the inter spacer region (ITS) region as described previously.^26^ Sequencing data were analyzed using the ITS SmartGene custom database, applying the identification guidelines by Ciardo et al.^27^

Antifungal susceptibility testing (AFST) was performed on request or if deemed clinically relevant by the treating physician. AFST was done by gradient diffusion strips using Etest (bioMérieux, France; Liofilchem S.r.I., Italy). Conidia from a well sporulated culture on PCA were resuspended in 0.85% NaCl with 0.01% Tween80. After 3–5 min of sedimentation, the supernatant, without the mycelium below, was transferred to a fresh tube and vortexed vigorously. A fungal suspension of McFarland 0.5–1 was prepared in fresh 0.85% NaCl and spread onto Roswell Park Memorial Institute (RPMI) agar plates (bioMérieux, France) with a cotton swab. Antifungal gradient strips were applied, and the plates were incubated at 35°C. Minimal inhibitory concentrations (MIC) were read after 24–48 h of incubation. MICs for amphotericin B were read at complete growth inhibition, MICs for azoles (voriconazole, posaconazole, isavuconazole, and itraconazole) and echinocandins (anidulafungin and caspofungin) were read at 80% of growth inhibition (minimal effective concentration). Micafungin AFST was performed by broth microdilution using the Sensititre YeastOne panel (TREK Diagnostic Systems, UK) according to the manufacturer's manual for *Aspergillus spp*. Etest MIC values were rounded up and adjusted to a binary log scale.

If several isolates per patient were tested, only the first isolate per patient per year and material were included in this study.

Statistical data were analyzed using SPSS version 29.0 (IBM Corp., USA) and R Version 2023.06.1 + 524. Baseline characteristics were calculated by frequency, average, or mean. Comparison analysis was performed by Fisher's exact test. A *P*-value of < 0.05 was considered statistically significant. Death, successful eradication, and strength of association with surgical debridement and antifungal therapy were analyzed by calculation of odds ratio, with determination of confidence interval.

## Results

### Patient characteristics

Patient's baseline characteristics are presented in Table 1. Out of 40 patients with positive microbiological samples for *Scedosporium* spp., 38 were included in the analysis. In the patients classified as having infections (*n* = 10), nine presented as localized infection and one as invasive infection (eye trauma with presence of hyphae in the surrounding periorbital tissue). After a polytrauma related to a traffic accident the infection was localized in the calf muscles. The majority of patients (*n* = 25; 65.7%) with a positive sample had underlying lung disease. Out of these 25 patients, 22 (88%) were classified as colonization and three (12%) as localized infection at initial diagnosis.

**Table 1.** Baseline characteristics of 38 patients with *Scedosporium* spp. detection in microbiological samples.

		*n* Total = 38	(%)	Colonization (*n* = 28; 73.7%)	Infection (*n* = 10; 26.3%)	Average	Mean	Minimum	Maximum	Odds ratio (95% CI)	*P*-value
Age at diagnosis (years)						47	53	19	77		
Gender	Female	20	52.6%	14	6					2.25 (0.3-17)	0.3
	Male	18	47.4%	14	4						
Underlying condition	Cystic fibrosis	12	31.6%	9	3					1.1 (0.2-8.2)	1
	Cystic fibrosis with lung TPL	4	10.5%	4	0					NA	0.55
	Lung TPL with other condition than CF †	3	7.9%	3	0					NA	0.55
	Chronic rhinosinusitis	1	2.6%	0	1					NA	0.26
	Chronic otitis media	2	5.3%	2	0					NA	1
	Bronchiectasis	6	15.8%	6	0					NA	0.179
	Eye infections ‡	4	10.5%	0	4					NA	<0.01
	Polytrauma	2	5.3%	1	1					0.34 (0-29.1)	0.46
	Other §	4	10.5%	3	1					1.1 (0–62.3)	1
immunosuppression	Yes	12	31.6%	11	1					5.6 (0.6-277.6)	0.12
	No	26	68.4%	17	9						

† Other conditions for lung TPL: pulmonary fibrosis, bronchiectasis, COPD

‡ Eye infections: exogenous endophthalmitis, contact lens associated keratitis, dacryocystitis, postoperative keratitis

§ Other: granulomatosis with polyangiitis, erythroderma with topical use of high dose steroids, aortic dissection, pulmonary *M. intracellulare* infection

### Diagnostic steps initiated after the initial detection

Upon evidence of a positive culture histological additional diagnostic investigations were performed in 10 out of 38 patients (26.3%), bronchoscopy in five out of 25 patients with pulmonary pathologies (20%), and imaging (e.g., CT scan of lungs or brain) in 12 patients (31.6%) (cf. Table 2). In the vast majority of patients (*n* = 28; 73.7%) only a single culture was positive, whereas in the remaining patients (*n* = 10; 26.3%) cultures were repetitively positive beyond 14 days of initial detection, ranging from five to 10 times over a time period from 24 to 138 days (mean 67 days).

**Table 2.** Microbiological diagnostics and results, further diagnostics at initial microbiological detection.

		*n*	(%)	Colonization	Infection	*P*-value
Microbiological result	*Scedosporium apiospermum* complex	23	60.5%	18	5	0.47
	*Scedosporium aurantiacum*	1	2.6%	1	0	1
	*Lomentospora prolificans*	0	0.0%			
	*Scedosporium* sp. †	14	36.8%	9	5	0.49
Diagnostic method	Culture	9	23.7%	8	1	0.4
	Culture and histopathology	2	5.3%	1	1	0.46
	Culture and molecular methods	27	71.1%	19	8	0.69
Material	Sputum	18	47.4%	16	2	0.07
	BAL	5	13.2%	5	0	0.30
	TBS	4	10.5%	4	0	0.56
	Tissue	7	18.4%	2	5	**<0.01**
	Corneal swab	2	5.3%	0	2	0.06
	Paranasal sinus fluid	1	2.6%	0	1	0.23
	Middle ear swab	1	2.6%	1	0	1
Histology	not performed	28	73.7%	24	6	0.17
	performed, but without evidence of fungal structures	9	23.7%	6	3	0.67
	histology showing fungal structures	1	2.6%	0	1	0.23
Intervention	no further intervention	28	73.7%	23	5	0.09
	Bronchoscopy	5	13.2%	3	2	059
	Biopsy	5	13.2%	2	2	0.28
Imaging	no	26	68.4%	21	5	0.24
	yes	12	31.6%	7	5	

† Assignment as *Scedosporium sp*. was applied when isolates were only identified by microscopy and phenotypic features or when sequencing did not allow unequivocal identification based on the used database. TBS: tracheobronchial secretion; BAL: bronchoalveolar lavage.

### Microbiology

For the final identification and to exclude *Lomentospora prolificans*, 27 (71.1%) isolates were analyzed by sequencing of the ITS region. A total of 23 isolates belonged to the *S. apiospermum* complex (including *S. boydii*), one isolate was identified as *S. aurantiacum*, and three isolates could only be identified to genus level, because sequencing of the ITS region cannot distinguish *Scedosporium dehoogii* from the *S. apiospermum* complex.^1^ The remaining 11 isolates were only identified by microscopy and phenotypic features.

Antifungal resistance patterns of 21 *Scedosporium* species isolated from 17 patients are shown in Table 3 and detailed in Appendix 1. Choice of antifungals tested varied, depending on consultation with clinicians. Furthermore, *Scedosporium* isolates from 10 patients were tested retrospectively on all shown antifungals, including micafungin, which was only available at our lab as part of the Sensititre YeastOne microdilution panel. All isolates were tested for voriconazole, which had the lowest MIC_90_ (inhibition of 90% of the isolates) of 0.125 µg/ml and an MIC range of 0.008–0.5 µg/ml, followed by posaconazole (*n* = 19) and isavuconazole (*n* = 14) both with an MIC_90_ of 1 µg/ml and MICs ranging from 0.016 to 2 µg/ml and 0.008 to 1 µg/ml, respectively. *In vitro* activity of amphotericin B (*n* = 18) against *Scedosporium* sp. was low and MICs ranged from 0.5 to 32 µg/ml with a median MIC (MIC_50_) of 8 µg/ml and a MIC_90_ of 32 µg/ml. Total numbers of *Scedosporium* sp. tested for echinocandins were low; caspofungin (*n* = 13), anidulafungin (*n* = 11), and micafungin (*n* = 10), and the echinocandins showed very low *in vitro* activity, with caspofungin being the most effective with MICs ranging from 0.064 to 32 µg/ml, an MIC_50_ of 1 µg/ml, and an MIC_90_ of 32 µg/ml. Micafungin showed no *in vitro* activity against *Scedosporium* sp.

**Table 3.** **Antibiotic resistance patterns of 21 *Scedosporium* spp. isolated from 17 patients**. In three patients with recurring or disseminated *Scedosporium* infections one AFST per year or specimen was included in the analyses.

					MIC (µg/ml)												
antifungal	MIC range	MIC_50_	MIC_90_	*n*	≤0.008	0.016	0.032	0.064	0.125	0.25	0.5	1	2	4	8	16	≥32
Amphotericin B	0.5–32	8	32	18							3	4		1	2		8
Voriconazole	0.008–0.5	0.064	0.125	21	2	2	6	6	4		1						
Posaconazole	0.016–2	0.25	1	19		1	1		5	5	3	3	1				
Isavuconazole	0.008–1	0.1875	1	14	1	1	2	2	1	2	4	1					
Itraconazole	0.008–2	0.5	2	15	1				3	1	4	2	4				
Caspofungin	0.064–32	1	32	13				1	1	1	3	2	1	1			3
Anidulafungin	0.5–32	32	32	11							1	1	1	2			6
Micafungin	8–8	8	8	10											10 †		

† Sensititre YeastOne echinocandine concentrations ranges only up to 8 µg/ml

### Clinical evolution and diagnostic evaluation after initial detection

Evolution from colonization into infection was observed in a total of two patients (2/28; 7.1%). One developed an invasive infection from pulmonary colonization after lung transplantation with a septic knee arthritis 88 days after first microbiological detection (while no antifungal therapy was initiated at diagnosis of colonization). The other patient with CF developed an allergic bronchopulmonal mycosis (ABPM) 501 days after first detection. Both infections were caused by the *S. apiospermum* complex.

Follow-up imaging at six and 12 weeks was performed in 10 patients with initial colonization (*n* = 5) or infection (*n* = 5). At week six, progression of infection was demonstrated in two patients (40%), while two had stable (40%) and one regressive findings (20%). At week 12, no patient had progression, four had stable (66.7%), and two had regressive findings (33.3%). Of note, some patients only underwent follow-up imaging at weeks 6 or 12.

### Treatment

**Figure 1.**
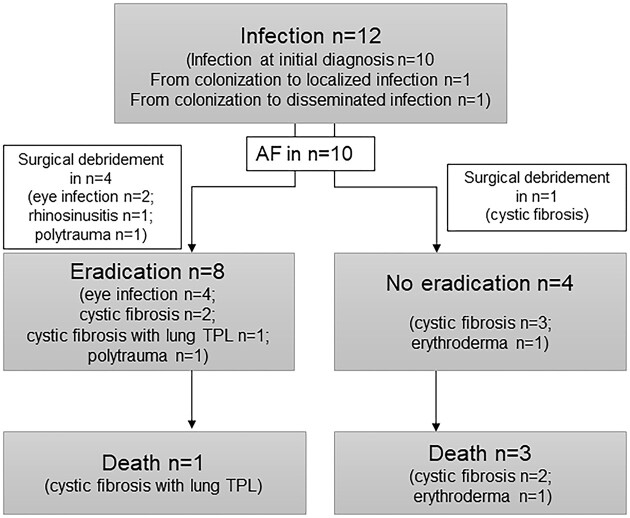
Outcome and interventions of initially or during observation time diagnosed infections with Scedosporium spp. Antifungal treatment (AF) was initiated in ten out of twelve patients, while in the remaining two patients (with chronic sinusitis resp. dacryocystitis) surgical debridement was considered sufficient for eradication.

At initial detection, antifungal treatment was initiated in eight out of 10 patients categorized as infection (80%) (Fig. 1). Two patients, both with CF, had a treatment delay of 15 and 52 days, respectively. In one patient, it was due to unawareness of microbiological results, while in the second patient, no treatment indication was made as inflammation parameters were absent. Two patients (dacryocystitis and chronic rhinosinusitis) had no antifungal treatment at all and only surgical debridement was performed.

Voriconazole was the main initial antifungal treatment (9/10; 90%). In one patient, a combination of voriconazole and terbinafine was initiated (based on physician's assessment upon the finding of a disseminated infection with septic knee arthritis). Three patients (30%) developed a voriconazole induced drug toxicity requiring a switch to posaconazole (*n* = 2) and isavuconazole (*n* = 1) as second-line treatment. Antifungal treatment could be stopped in five patients (50%) due to regression of initial findings. If treatment could be stopped, it lasted for a median time of 98.5 days [SD 66 days; eye infections (*n* = 3), ABPM (*n* = 1); polytrauma (*n* = 1)]. In the remaining five patients (50%) in whom treatment could not be stopped, treatment duration lasted until day of death (11–1168 days, median: 573 days) or is still ongoing at last follow-up.

Surgical debridement was performed in five patients, including patients with eye infections (*n* = 2), septic knee arthritis (*n* = 1), polytrauma (*n* = 1), and chronic rhinosinusitis (*n* = 1). In the remaining five patients, only antifungal treatment was administered [cystic fibrosis (*n* = 3), contact lens-associated and postoperative keratitis (*n* = 2)]. Concomitant reduction of immunosuppressive was not conducted in any of the two patients with infection.

### Outcome

The overall mortality rate of patients with microbiological detection of *Scedosporium* spp. infection was 31.6% (12/38). Patients diagnosed with infection had a mortality rate of 33.3% (4/12) compared to 30.7% (8/26) (*P* = 1.0) in the group with colonization. Patients under immunosuppressive therapy had a mortality rate of 50% (6/12), with an equal 50% mortality rate in the group with infection (1/2) and colonization (5/10). Patients without any immunosuppressive therapy had a mortality rate of 30% (3/10) with infection and 18.8% (3/16) (*P* = 0.64) with colonization.

Median time to death was 540 days after first microbiological detection in infection (12–1201, SD 489 days) and 691 days in colonization (62–1440; SD 466 days). Eradication of initial infection could be achieved in eight out of 12 patients (66.6%) (Fig. 1). In the group with successful eradication, death occurred in one out of eight patients (12.5%) compared to three out of four patients (75%), where no eradication was achieved (OR 21.0; CI 0.96-458.86; *P* = 0.053). Only in one patient (lung transplantation due to CF with disseminated *S. apiospermum* infection) death was related to the mould infection, while in the other three patients causes of death were unrelated.

Surgical debridement was performed in four out of eight (50%) patients with successful eradication (two patients were concomitantly treated with antifungals) and in one out of four (25%) patients without eradication (OR 3.0; CI 0.21-42.62; *p* = 0.58). No death occurred in patients undergoing surgical debridement.

Of the 10 patients receiving antifungal treatment, four patients died (40%), including one patient receiving a combination of voriconazole and terbinafine. Eradication was achieved in six patients (60%) after antifungal treatment. No eradication could be achieved in the two patients with CF where treatment initiation was delayed.

## Discussion

Lung as the main site of infection is in line with reports from earlier cohort studies.^13,28–30^ The second most frequent sites were ocular infections, also described in previous studies.^22^ Some frequent sites of infections, such as bone and brain, typically associated with disseminated infections, are missing in our study altogether.^29–31^ Only one out of 12 infections (8.3%) progressed to disseminated disease. This is less than described in the literature, where rates of dissemination ranged from 11.5% up to 30%. The low prevalence of severely immunocompromised patients (specifically, no neutropenic patient nor after HSCT) may be one of the main factors. Other explanations for this low rate of disseminated disease might be the intensified prevention strategies for mould infections in the last two decades, including mould-specific antifungal prophylaxis after solid organ transplantation with activity against *Scedosporium* spp. (but limited activity against *Lomentospora prolificans*).

Upon microbiological detection, further diagnostic steps were limited to a minority of patients and not following recommendations from current guidelines with the goal to differentiate between colonization and infection, or to assess the extent of infection.^18,21^ Only one-third of all patients underwent imaging modalities to assess for signs of invasive mould infections, even though imaging is strongly encouraged by different recommendations.^4,18^ Histologic examinations yielded an invasive infection in one out of 10 biopsies, (endophthalmitis with infiltration of periorbital tissue). Despite this low yield, exclusion of an invasive disease can guide further management. One possible explanation for this very low adherence to guidelines is that the limited awareness about the potential danger does not trigger further examinations.

Surgical debridement represents one of the cornerstones in the initial management in localized lesions.^7^ In our population, it was performed in five out of 12 patients with infection (41.6%). In patients experiencing surgical debridement, no death was observed during the observation period. Small numbers preclude any strong recommendations. While certain case series did not demonstrate an improvement in overall survival following surgery, others showed an association with decreased mortality rates, thus limiting general conclusions regarding the impact of surgical debridement on mortality.^22,31^

Reduction of immunosuppression, a further cornerstone in treatment of mould infections, was not performed in the two patients with infection under immunosuppressive treatment. Awareness of this management strategy should be reinforced using an interdisciplinary approach whenever this reduction can be taken into consideration. In two cases, of which one with immunosuppression, treatment was delayed despite diagnosis of infection, which represents an independent predictor for mortality at 3 months.^30^ This further supports the importance of an infectious disease consultation upon microbiological detection.

As expected, the antifungal susceptibility testing results were concordant to published results with lowest MIC for voriconazole followed by posaconazole.^24,32^ Despite the weak recommendation supporting its usage in the treatment of scedosporiosis, isavuconazole was administered in one patient as a second-line treatment due to drug toxicity with voriconazole.^18^ In general, AFST should not primarily guide the choice of antifungal treatment and is only recommended with moderate evidence as clinical breakpoints are missing.^9^ However, it can help choosing a second-line antifungal treatment as in this case. Combination therapy with terbinafine and azoles, as applied as first-line treatment in one patient, exhibits an in vitro synergistic activity.^34,35^ Due to poor tissue penetration of terbinafine and scarce clinical data on outcome, the clinical significance of this combination remains unclear and is moderately recommended as first-line alternative over voriconazole monotherapy.^18,24^

It remains unclear why certain patients progress from colonization to infection, as this evolution can be observed in immunocompetent and immunocompromised patients. Furthermore, infections can still be observed despite antifungal prophylaxis, especially in *Scedosporium* spp. colonization after lung transplantation, under life-long antifungal prophylaxis.^15^ A very recent study included colonization in their cohort, but no information was provided on the clinical evolution.^29^ Other cohorts actively excluded colonization or were focused on patients with colonization in CF or lung transplant recipients only.^22,30–34^

The overall mortality rate was similar in the colonization and infection groups (30.7% and 33.3%, respectively) and higher in the subgroups receiving immunosuppressive therapy (50% in colonization and infection) compared to immunocompetent patients (30% in infection, 18.8% in colonization). In a very recent study, airway colonization with *Scedosporium* spp. in CF patients has been associated with higher numbers of hospitalizations and bacterial infections as compared to CF patients without colonization.^35^ Infection with *S. apiospermum* complex was directly attributed to scedosporiosis in only one patient of our cohort. Published series described infection attributed mortality of up to 76%.^22,36^ Overall mortality from these large cohorts ranged from 43.6% to 70% in immunocompromised and 30.7% in immunocompetent patients.^22,30,37^ Here again, our population is biased by the fact, that only few patients were immunocompromised, had disseminated disease or central nervous system involvement, which are all representing predictors for mortality.^22,31^

Assessment of eradication of infection is representing a new aspect for evaluating *Scedosporium* spp. infections outside the field of CF or lung transplantation, where especially eradication of colonization by antifungal treatment has been studied, but mostly with poor results.^38,39^ In this cohort, even though eradication and surgical debridement were associated with a lower number of deaths, a major bias is represented by the relatively high numbers of ocular infections, corresponding to a less comorbid patient population than patients with pulmonary pathologies.

One of the study's main limitations, next to the already above-mentioned biases, is the small and very heterogeneous cohort size. This restricts the interpretation for significant results especially regarding outcome data or the performance of a multivariate analysis. Thus, no general conclusions can be drawn and studies in larger multicentric cohorts are warranted, especially to explore risk factors for clinical evolution from colonization to infection.

*Scedosporium* spp. colonization and infections are representing a major marker for morbidity and mortality. As the management upon microbiological detection is complex and can directly influence patient's outcome, a multidisciplinary approach should be encouraged in particular to define further diagnostics and treatment options.

## Supplementary Material

myae002_Supplemental_File

## References

[1] Ramirez-Garcia A , Pellon A, Rementeria A et al. Scedosporium and Lomentospora: an updated overview of underrated opportunists. Med Mycol. 2018; 56(suppl_1): 102–125. 29538735 10.1093/mmy/myx113

[2] Lackner M , de Hoog GS, Yang L et al. Proposed nomenclature for Pseudallescheria, Scedosporium and related genera. Fungal Diversity. 2014; 67: 1–10.

[3] Mello TP , Bittencourt VCB, Liporagi-Lopes LC, Aor AC, Branquinha MH, Santos ALS. Insights into the social life and obscure side of Scedosporium/Lomentospora species: ubiquitous, emerging and multidrug-resistant opportunistic pathogens. Fung Biol Rev. 2019; 33: 16–46.

[4] Husain S , Muñoz P, Forrest G et al. Infections due to *Scedosporium apiospermum* and *Scedosporium prolificans* in transplant recipients: clinical characteristics and impact of antifungal agent therapy on outcome. Clin Infect Dis. 2005; 40: 89–99. 15614697 10.1086/426445

[5] Thomas PA , Kaliamurthy J. Mycotic keratitis: epidemiology, diagnosis and management. Clin Microbiol Infect. 2013; 19: 210–220. 23398543 10.1111/1469-0691.12126

[6] Dinh A , Demay O, Rottman M et al. Case of femoral pseudarthrosis due to *Scedosporium apiospermum* in an immunocompetent patient with successful conservative treatment and review of literature. Mycoses. 2018; 61: 400–409. 29274090 10.1111/myc.12739

[7] Tortorano AM , Richardson M, Roilides E et al. ESCMID and ECMM joint guidelines on diagnosis and management of hyalohyphomycosis: Fusarium spp., Scedosporium spp. And others. Clin Microbiol Infect. 2014; 20(Suppl 3): 27–46. 10.1111/1469-0691.12465 24548001

[8] Bouchara JP , Papon N. Scedosporium apiospermum. Trends Microbiol. 2019; 27: 1045–1046. 31378439 10.1016/j.tim.2019.07.003

[9] Rammaert B , Puyade M, Cornely OA et al. Perspectives on *Scedosporium* species and *Lomentospora prolificans* in lung transplantation: results of an international practice survey from ESCMID fungal infection study group and study group for infections in compromised hosts, and European Confederation of Medical Mycology. Transpl Infect Dis. 2019; 21: e13141. 31283872 10.1111/tid.13141

[10] Cortez KJ , Roilides E, Quiroz-Telles F et al. Infections caused by Scedosporium spp. Clin Microbiol Rev. 2008; 21:157–197. 18202441 10.1128/CMR.00039-07 PMC2223844

[11] Rodriguez-Tudela JL , Berenguer J, Guarro J et al. Epidemiology and outcome of *Scedosporium prolificans* infection, a review of 162 cases. Med Mycol. 2009; 47: 359–370. 19031336 10.1080/13693780802524506

[12] Lass-Flörl C , Cuenca-Estrella M. Changes in the epidemiological landscape of invasive mould infections and disease. J Antimicrob Chemother. 2017; 72(suppl_1): i5–i11. 28355462 10.1093/jac/dkx028

[13] Heath CH , Slavin MA, Sorrell TC et al. Population-based surveillance for scedosporiosis in Australia: epidemiology, disease manifestations and emergence of *Scedosporium aurantiacum* infection. Clin Microbiol Infect. 2009; 15: 689–693. 19549223 10.1111/j.1469-0691.2009.02802.x

[14] Johnson LS , Shields RK, Clancy CJ. Epidemiology, clinical manifestations, and outcomes of Scedosporium infections among solid organ transplant recipients. Transpl Infect Dis. 2014; 16: 578–587. 24962102 10.1111/tid.12244

[15] Parize P , Boussaud V, Poinsignon V et al. Clinical outcome of cystic fibrosis patients colonized by Scedosporium species following lung transplantation: a single-center 15-year experience. Transpl Infect Dis. 2017; 19. 10.1111/tid.12738 28618155

[16] Hoenigl M , Salmanton-García J, Walsh TJ et al. Global guideline for the diagnosis and management of rare mould infections: an initiative of the European Confederation of Medical Mycology in cooperation with the International Society for Human and Animal Mycology and the American Society for Microbiology. Lancet Infect Dis. 2021; 21: e246–e57. 33606997 10.1016/S1473-3099(20)30784-2

[17] Shoham S , Dominguez EA. Emerging fungal infections in solid organ transplant recipients: Guidelines of the American Society of Transplantation Infectious Diseases Community of Practice. Clin Transplant. 2019; 33: e13525. 30859651 10.1111/ctr.13525

[18] Stemler J , Lackner M, Chen SC, Hoenigl M, Cornely OA. EQUAL score Scedosporiosis/Lomentosporiosis 2021: a European Confederation of Medical Mycology (ECMM) tool to quantify guideline adherence. J Antimicrob Chemother. 2021; 77: 253–258. 34542613 10.1093/jac/dkab355 PMC8730684

[19] Abela IA , Murer C, Schuurmans MM et al. A cluster of scedosporiosis in lung transplant candidates and recipients: the Zurich experience and review of the literature. Transpl Infect Dis. 2018; 20. 10.1111/tid.12792 29044831

[20] Khoueir N , Verillaud B, Herman P. *Scedosporium apiospermum* invasive sinusitis presenting as extradural abscess. Eur Ann Otorhinolaryngol Head Neck Dis. 2019; 136: 119–121. 30528155 10.1016/j.anorl.2018.11.009

[21] Donnelly JP , Chen SC, Kauffman CA et al. Revision and update of the consensus definitions of invasive fungal disease from the European Organization for Research and Treatment of Cancer and the Mycoses Study Group Education and Research Consortium. Clin Infect Dis. 2020; 71: 1367–1376. 31802125 10.1093/cid/ciz1008 PMC7486838

[22] Seidel D , Meißner A, Lackner M et al. Prognostic factors in 264 adults with invasive scedosporium spp. And lomentospora prolificans infection reported in the literature and FungiScope(®). Crit Rev Microbiol. 2019; 45: 1–21. 30628529 10.1080/1040841X.2018.1514366

[23] Segal BH , Herbrecht R, Stevens DA et al. Defining responses to therapy and study outcomes in clinical trials of invasive fungal diseases: Mycoses Study Group and European Organization for Research and Treatment of Cancer consensus criteria. Clin Infect Dis. 2008; 47: 674–683. 18637757 10.1086/590566 PMC2671230

[24] Ciardo DE , Lucke K, Imhof A, Bloemberg GV, Böttger EC. Systematic internal transcribed spacer sequence analysis for identification of clinical mold isolates in diagnostic mycology: a 5-year study. J Clin Microbiol. 2010; 48: 2809–2813. 20573873 10.1128/JCM.00289-10 PMC2916574

[25] de Hoog GS , Gené J, Figueras MJ. Atlas of Clinical Fungi, 3rd edition ed. Hilversum: Westerdijk Institute / Universitat Rovira i Virgili, Utrecht / Reus, 2009.

[26] Rampini SK , Zbinden A, Speck RF, Bloemberg GV. Similar efficacy of broad-range ITS PCR and conventional fungal culture for diagnosing fungal infections in non-immunocompromised patients. BMC Microbiol. 2016; 16: 132. 27349889 10.1186/s12866-016-0752-1 PMC4924236

[27] Ciardo DE , Schär G, Böttger EC, Altwegg M, Bosshard PP. Internal transcribed spacer sequencing versus biochemical profiling for identification of medically important yeasts. J Clin Microbiol. 2006; 44: 77–84. 16390952 10.1128/JCM.44.1.77-84.2006 PMC1351945

[28] Rougeron A , Giraud S, Alastruey-Izquierdo A et al. Ecology of Scedosporium species: present knowledge and future research. Mycopathologia. 2018; 183: 185–200. 28929280 10.1007/s11046-017-0200-2

[29] Lao CK , Ou JH, Fan YC, Wu TS, Sun PL. Clinical manifestations and susceptibility of Scedosporium/Lomentospora infections at a tertiary medical centre in Taiwan from 2014 to 2021: a retrospective cohort study. Mycoses. 2023; 66: 923–935. 37449538 10.1111/myc.13632

[30] Bronnimann D , Garcia-Hermoso D, Dromer F, Lanternier F. Scedosporiosis/lomentosporiosis observational study (SOS): clinical significance of scedosporium species identification. Med Mycol. 2021; 59: 486–497. 33037432 10.1093/mmy/myaa086

[31] Neoh CF , Chen SCA, Crowe A et al. Invasive *Scedosporium* and *Lomentospora prolificans* infections in Australia: a multicenter retrospective cohort study. Open Forum Infect Dis. 2023; 10: ofad059. 36861090 10.1093/ofid/ofad059 PMC9970007

[32] Erro Iribarren M , Girón Moreno RM, Diab Cáceres L et al. Study of a cohort of patients with cystic fibrosis and isolation of Scedosporium spp. Arch Bronconeumol (Engl Ed). 2019; 55: 559–564. 31178266 10.1016/j.arbres.2019.02.018

[33] Vazirani J , Westall GP, Snell GI, Morrissey CO. *Scedosporium apiospermum* and *Lomentospora prolificans* in lung transplant patients—a single center experience over 24 years. Transpl Infect Dis. 2021; 23: e13546. 33315292 10.1111/tid.13546

[34] Schwarz C , Brandt C, Antweiler E et al. Prospective multicenter German study on pulmonary colonization with Scedosporium/Lomentospora species in cystic fibrosis: epidemiology and new association factors. PLoS One. 2017; 12: e0171485. 28178337 10.1371/journal.pone.0171485 PMC5298894

[35] Parize P , Fleury M, Poupon-Bourdy S et al. Outcome of patients with cystic fibrosis colonized by Scedosporium and Lomentospora species: a longitudinal cohort study. Med Mycol. 2023; 61. 10.1093/mmy/myad051 37263788

[36] Heng SC , Slavin MA, Chen SC et al. Hospital costs, length of stay and mortality attributable to invasive scedosporiosis in haematology patients. J Antimicrob Chemother. 2012; 67:2274–2282. 22643193 10.1093/jac/dks210

[37] Lamaris GA , Chamilos G, Lewis RE, Safdar A, Raad II, Kontoyiannis DP. Scedosporium infection in a tertiary care cancer center: a review of 25 cases from 1989–2006. Clin Infect Dis. 2006; 43: 1580–1584. 17109292 10.1086/509579

[38] Bentley S , Davies JC, Carr SB, Balfour-Lynn IM. Combination antifungal therapy for scedosporium species in cystic fibrosis. Pediatr Pulmonol. 2020; 55: 1993–1995. 32339450 10.1002/ppul.24789

[39] Tamm M , Malouf M, Glanville A. Pulmonary scedosporium infection following lung transplantation. Transpl Infect Dis. 2001;3: 189–194. 11844150 10.1034/j.1399-3062.2001.30402.x

