# Supplementary material for: Detection of Scedosporium spp.: Colonizer or pathogen? A retrospective analysis of clinical significance and management in a large tertiary center
Source: Med Mycol. 2024 Jan 18;62(2):myae002. doi: 10.1093/mmy/myae002 (PMC10837104; doi:10.1093/mmy/myae002)
Supplement: myae002_Supplemental_File [file myae002_supplemental_file.docx]

**Appendix 1:**

**Antifungal susceptibility testing (AFST) per patient and year**

| ID | species |  | MIC $\mu$g/ml | | | | | | | |
| --- | --- | --- | --- | --- | --- | --- | --- | --- | --- | --- |
|  |  | year | Voriconazole | Posaconazole | Isavuconazole | Itraconazole | Amphotericin B | Anidulafungin | Caspofungin | Micafungin |
| 37 | *Scedosporium* sp | 2020 | 0.032 | 0.032 |  |  |  |  |  |  |
| 11 | *S. apiospermum* complex | 2015 | 0.032 | 0.125 |  | 0.125 | 1 | 2 | 1 |  |
| 11 | *S. apiospermum* complex | 2017 | 0.016 | 0.125 | 0.032 | 0.125 | 1 | 32 | 0.064 | 8 |
| 10 | *S. apiospermum* complex | 2017 | 0.125 | 0.125 | 0.032 | 2 | 4 | 32 | 1 | 8 |
| 39 | *Scedosporium* sp | 2021 | 0.064 |  | 0.5 |  |  |  |  |  |
| 7 | *S. apiospermum* complex | 2016 | 0.032 | 0.25 | 0.064 | 0.5 | 1 | 4 | 4 | 8 |
| 7 | *S. apiospermum* complex | 2017 | 0.016 | 0.5 |  | 0.5 | 0.5 |  |  |  |
| 7 | *S. apiospermum* complex | 2018 | 0.125 | 1 | 0.5 |  | 0.5 |  | 0.5 |  |
| 19 | *Scedosporium* sp | 2018 | 0.125 |  |  | 2 | 8 |  |  |  |
| 2 | *S. aurantiacum* | 2015 | 0.064 | 0.5 | 0.25 | 2 | 32 | 0.5 | 32 | 8 |
| 3 | *S. apiospermum* complex | 2016 | 0.032 | 0.25 | 0.25 | 1 | 32 | 32 | 0.5 | 8 |
| 6 | *S. apiospermum* complex | 2016 | 0.008 | 0.016 | 0.008 | 0.008 | 32 | 4 | 0.25 | 8 |
| 13 | *S. apiospermum* complex | 2016 | 0.032 | 0.125 |  | 0.125 | 0.5 |  |  |  |
| 4 | *S. apiospermum* complex | 2015 | 0.032 | 0.25 | 0.016 | 1 | 1 | 32 | 32 | 8 |
| 15 | *S. apiospermum* complex | 2016 | 0.5 | 0.125 |  |  |  |  |  |  |
| 1 | *S. apiospermum* complex | 2015 | 0.064 | 1 |  | 2 | 32 |  |  |  |
| 1 | *S. apiospermum* complex | 2016 | 0.064 | 0.25 | 0.5 | 0.5 | 32 | 32 | 32 | 8 |
| 5 | *S. apiospermum* complex | 2016 | 0.064 | 1 | 0.064 | 0.25 | 8 | 32 | 0.125 | 8 |
| 27 | *Scedosporium* sp | 2019 | 0.064 | 2 | 1 |  | 32 |  |  |  |
| 38 | *S. apiospermum* complex | 2021 | 0.125 | 0.5 | 0.5 |  | 32 |  | 0.5 |  |
| 8 | *S. apiospermum* complex | 2016 | 0.008 | 0.25 | 0.125 | 0.5 | 32 | 1 | 2 | 8 |
